# Transcriptomics Analysis and Re-sequencing Reveal the Mechanism Underlying the Thermotolerance of an Artificial Selection Population of the Pacific Oyster

**DOI:** 10.3389/fphys.2021.663023

**Published:** 2021-04-22

**Authors:** Yulong Tan, Rihao Cong, Haigang Qi, Luping Wang, Guofan Zhang, Ying Pan, Li Li

**Affiliations:** ^1^College of Animal Science and Technology, Guangxi University, Nanning, China; ^2^CAS and Shandong Province Key Laboratory of Experimental Marine Biology, Center for Ocean Mega-Science, Institute of Oceanology, Chinese Academy of Sciences, Qingdao, China; ^3^Laboratory for Marine Biology and Biotechnology, Pilot National Laboratory for Marine Science and Technology, Qingdao, China; ^4^Laboratory for Marine Fisheries Science and Food Production Processes, Pilot National Laboratory for Marine Science and Technology, Qingdao, China; ^5^Center for Ocean Mega-Science, Chinese Academy of Sciences, Qingdao, China

**Keywords:** Pacific oyster, artificial selection, thermotolerance, constitutive difference of gene expression, gene structure

## Abstract

The Pacific oyster is a globally important aquaculture species inhabiting the intertidal environment, which experiences great temperature variation. Mass deaths in the summer pose a major challenge for the oyster industry. We initiated an artificial selection breeding program in 2017 using acute heat shock treatments of the parents to select for thermotolerance in oysters. In this study, we compared the respiration rate, summer survival rate, gene expression, and gene structure of F_2_ selected oysters and non-selected wild oysters. A transcriptional analysis revealed global divergence between the selected and control groups at the larval stage, including 4764 differentially expressed genes, among which 79 genes were heat-responsive genes. Five heat shock proteins were enriched, and four of the six genes (five heat stock genes in the enriched GO terms and KEGG pathways and *BAG4*) were differentially expressed in 1-year-old oysters. Integration of the transcriptomic and re-sequencing data of the selected and the control groups revealed 1090 genes that differentiated in both gene structure and expression. Two SNPs (single nucleotide polymorphism) that may mediate the expression of *CGI_10022585* and *CGI_10024709* were validated. In addition, the respiration rate of 1-year-old oysters varied significantly between the selected group and the control group at room temperature (20°C). And the summer survival rate of the selected population was significantly improved. This study not only shows that artificial selection has a significant effect on the gene structure and expression of oysters, but it also helps reveal the mechanism underlying their tolerance of high temperature as well as the ability of oysters to adapt to climate change.

## Introduction

The Pacific oyster, *Crassostrea gigas* (Bivalvia: Ostreidae), is native to the Northwest Pacific Ocean and is widely distributed along the coasts of Japan, Korea, and northern China. Because of its adaptability and rapid growth (Guo, 2009), it has become a globally important aquaculture species. In 2018, the breeding area of the Pacific oyster spanned 144,000 hectares, and its production was 5.14 million tons, accounting for 36% of the total production of marine shellfish aquaculture in China.

Mass mortality of the Pacific oyster in summer has been observed in many countries since the middle of the 20th century. The mass mortality at different development stage was reported worldwide. For example, in the summer of 1992 and 1993, the Pacific oysters from several hatcheries in France had a high mortality rate (90–100%). Feeding and swimming ability declined 3–4 days after spawning, and a high mortality rate was observed on the 6th day after spawning. Most larvae died on the 8th to 10th day, when the mortality rate reached 100% (Tristan et al., 1994a). Mass deaths of Pacific oysters aged 3–7 months, with a mortality rate of 80–90%, were first reported in July 1993 along the Atlantic coast of France (Tristan et al., 1994b). The mass mortality of Pacific oysters poses a major challenge for the Chinese oyster industry. Large numbers of Pacific oysters in China have died because of high temperatures during the summer months since 1994. From 1995 to 1996, the mortality rate of individuals older than the second age class in the coastal areas of Dalian and Shandong reached 40–50%, and even 70–80%, in some areas. The individuals that survived also showed slow growth, which led to low production (Ma Shaosai et al., 1997; Shui et al., 1997).

Previous studies have suggested that environmental parameters, the physiological and genetic status of oysters, and the synergistic effects of pathogenic microorganisms may contribute to summer mortality (Tomaru et al., 2001; de Lorgeril et al., 2011; Genard et al., 2012). Seawater temperature, the amount of food, sea level atmospheric pressure, rainfall, and wind speed have all been associated with mortality risk (Fleury et al., 2020). Among all of the abiotic and biological factors that cause oyster mass mortality in summer, temperature rise is considered the most important (Le Roux et al., 2002; Gay et al., 2004; Meng et al., 2014). When exposed to heat stress, organisms adapt by increasing their metabolism and producing more energy (Sokolova et al., 2012). The respiration rate of winged pearl oyster was significantly higher under 30°C than under room temperature (Gu et al., 2020). In previous studies, heat stress has been shown to cause significant changes in the expression of heat shock proteins (HSPs) in oysters. HSPs are highly associated with thermotolerance, as heat shock induces the expression of HSP genes to mediate adaptation to high temperature (Zhang et al., 2012; Zhu et al., 2015; Liu Y. et al., 2019; Ding et al., 2020).

Heritability for summer survival rate was high, which suggests that selective breeding could effectively improve the thermotolerance of Pacific oysters (Dégremont et al., 2007). Previous studies have also shown that the mortality rate of oysters in summer can be significantly reduced in the offspring after artificial selection for thermotolerance. Specifically, the mortality rate of the selected population after 42 days was 19.6–43.4%, and the mortality rate of the control population after 42 days was 18–82%. This result has also been verified in the second generation, which had an average mortality rate of 19.6% during summer (Dégremont et al., 2010). In 2017, we launched an artificial selection and breeding program with the goal of improving summer survival rates. The selected F_1_ oysters had a significantly higher survival rate during the recovery period following heat shock at 42°C for 1 h compared with control F_1_ oysters. The survival rate of the 19-month-old selected population was significantly higher than that of the control population during summer under field conditions (Ding et al., 2020).

Populations that differ in their ability to resist environmental stress have been shown to differ in gene structure. For example, our previous study showed that the selected population and the control population differed in population structure by a PCA analysis based on SLAF sequencing (Ding et al., 2020). Populations of many species with different sensitivities to environmental stress often show large differences in gene expression (Kelly, 2019). In some studies, tolerant populations show greater differential gene expression between treatments [*Oncorhynchus mykiss*, *Salmo trutta* (Meier et al., 2014; Narum and Campbell, 2015), and *Gasterosteus aculeatus* (Morris et al., 2014)], whereas in other studies, sensitive populations show greater differential gene expression between treatments [*Tigriopus californicus* (Schoville et al., 2012), *Acropora hyacinthus* (Barshis et al., 2013), and *Chlorostoma funebralis* (Gleason and Burton, 2015)]. Pacific Oyster Mortality Syndrome (POMS)-resistant and sensitive oysters determined by field infection tests had differentially expressed genes (DEGs) involved in various pathways, which suggested that the resistance process is polygenic and varies with genotype (de Lorgeril et al., 2020). The association between various genotypes and gene expression provides further insight into the dissection of the different traits (Liu S. et al., 2019).

With the development of omics technology, transcriptomics and whole-genome re-sequencing have become increasingly used to identify differences in population structure and gene expression at the genome level. High-resolution melting and SNaPshot, which have medium or small throughput, are ne cost-effective for screening the SNPs (single nucleotide polymorphism) of a target region is (Zhang et al., 2020). We previously studied differentiation in genomic structure using the F_1_ generation of the selected and control groups for the thermotolerance. Here, we conducted a transcriptomic comparison of the F_2_ selected group and the control group at the larval stage. We investigated the constitutive gene expression of the second generation of thermotolerance oysters to investigate transcriptomic patterns and genomic differentiation after artificial selection. In addition, we integrate genotype and gene expression information at the adult stage to identify the genes involved in thermotolerance throughout development. We also identified potential genotypes that may regulate the thermotolerance by mediating gene expression. In addition, the reliability of artificial selection was verified by estimating the respiration rate and summer survival rate. Generally, this study provides key insights that could aid future molecular breeding efforts.

## Materials and Methods

### Sample Collection

Umbo larvae of the F_2_ of the selected group and the control group were used for transcriptomics and re-sequencing analysis, and 1-year-old oysters were used for the gene expression verification experiment.

In 2017, wild Pacific oysters were collected (the number of individuals in each population is greater than 300) in Shentanggou, Qingdao, Shandong (36° 21′ N, 120° 41′ E). One group was used as the parents of the control group, which was further cultured without any treatment; the other group was exposed to 42°C heat shock for 1 h, and the survivors were used as the parents of the thermotolerance group. We used 30 males and 30 females to produce the F_1_ and F_2_ of the selected and control group, respectively. Specifically, the eggs of the 30 females were mixed and equally divided into 30 beakers, each of which was fertilized by sperm from each of the 30 males. In 2018, thermotolerance was studied in the selected and control populations after a generation of artificial selection (Ding et al., 2020). In 2019, umbo larvae of the selected group, which were subjected to two generations of acute heat stress selection were collected and frozen in liquid nitrogen for subsequent experiments in a hatchery located in Laizhou, Yantai, Shandong Province. In 2020, 1-year-old oysters were used for respiration rate tests and gene expression tests after heat shock.

### Transcriptomics Analysis

Larvae of the selected group and the control group were used to extract total RNA; there were three replicates for each group. Total RNA was extracted by Trizol; RNA quality and concentration were measured by 1.0% agarose gel electrophoresis and UV spectrometry on a NanoDrop 2000 device, respectively. The transcriptome sequencing was performed on the Illumina HiSeq Xten platform. RNA-seq was conducted to investigate the expression differences between the selected and control oysters. After total RNA was isolated from the larvae in each group, it was first quantified and qualified by a Nanodrop 2000 device (Thermo Scientific). The library was constructed using the qualified samples based on the following procedures: (1) mRNAs were first enriched with magnetic beads containing Oligo(dT) and were randomly fragmented by fragmentation buffer; (2) the first cDNA strand was synthesized with random hexamers, the second cDNA strand was synthesized by adding buffer dNTPs, RNase H, and DNA polymerase I, and AMPure XP Beads were used to purify cDNA; (3) purified double-stranded cDNA was then repaired at the end, and a tail and connected sequencing adapters were added. AMPure XP beads were used to select the fragment size, and the cDNA library was finally obtained through PCR enrichment. The data were filtered using the following quality criteria: (1) reads containing adaptors were removed, and (2) low-quality reads were removed (i.e., reads with *N* > 10%, and reads in which *Q* ≤ 10 accounted for more than 50% of the bases of an entire read). The high-quality clean data were in FASTQ format following quality control. The reads were assembled and quantified using StringTie after sequence alignment analysis.

### Whole-Genome Re-sequencing Analysis

In November 2019, the larvae of the thermotolerance group and the control group were mixed to extract gDNA. gDNA was extracted by a TIANamp Marine Animals DNA Kit (Tiangen, Beijing, China); DNA quality and concentration were measured by 1.0% agarose gel electrophoresis and UV spectrometry on a NanoDrop 2000 device, respectively.

To study differences in SNP frequency and differences in the gene expression of SNPs, larvae of the thermotolerance group and the control group were selected for whole-genome re-sequencing. The qualified gDNA was fragmented by ultrasonic treatment; the DNA fragments were then purified and end-repaired, and the adaptors were added. The fragments were selected by agarose gel electrophoresis, and PCR was performed for enrichment. The produced library was sequenced with the Illumina HiSeq Xten platform. Raw reads with low quality were filtered out based on the following criteria: (1) reads containing adaptors were removed, (2) reads in which *N* > 10% were removed, and (3) reads in which *Q* < 10 for over 50% of the bases were removed. The reads were mapped to the reference genome, and variation analysis was performed. BWA (Li and Durbin, 2009) software was used to compare short sequences with the reference genome, and the location of clean reads on the reference genome was compared. Furthermore, the sequencing depth, genome coverage, and other information for each sample were determined, and the variation was detected.

The Euclidean distance (ED) algorithm was used to determine SNP frequency differences. ED can be used to find markers with significant differences between DNA pools associated with different traits (Geng et al., 2016; Yao et al., 2017). Regions with large ED values tend to be associated with the target traits of the two sample pools. After calculating ED for all SNPs, sites in which ED < 0.3 were removed.

### qPCR Validation in Adult Oysters

To determine whether the DEGs detected in larvae were also differentially expressed in adults, dominant genotypes associated with high resistance to heat stress were verified in adults. We assessed SNP frequency differences and identified the genes corresponding to the SNP loci from DEGs using RNA-seq. The 1-year-old oysters from the selected group and the control group were raised in seawater for 14 days. The oysters were exposed to heat stress at 35°C for 24 h and were sampled at 0, 12, and 24 h after heat shock, and 30 individuals were sampled at each time point. Total RNA quality was assessed using the same procedures described in section “Transcriptomics Analysis.”

Total RNA (0.5 μg) was reverse-transcribed into cDNA using the Evo M-MLV RT Kit with gDNA Clean for qPCR II. The cDNA was diluted 20 times to detect the gene expression level. The candidate genes were analyzed by qPCR. EF1-α was used as an internal reference gene. Primers for each gene were designed using Primer Premier 5.0 software (Supplementary Figure 1); qPCR was performed using the ABI7500 Fast Real-Time Detection System (Applied Biosystems, Foster City, CA, United States). The total volume of the reaction system was 20 μL and consisted of 10 μL of 2 × SYBR Green Pro TaqHS Premix (AG), 6.8 μL RNase-free water, 0.4 μL of each primer pair and ROX reference dye, and 2 μL of diluted cDNA. The program started with a 30-s activation of DNA polymerase at 95°C, followed by 40 cycles of 5 s at 95°C and 30 s at 60°C. The melt curve stage was as follows: 15 s at 95°C, 1 min at 60°C, 30 s at 95°C, and 15 s at 60°C. The expression level of the genes of induced transcripts was calculated using the Livak 2^–ΔΔCT^ method (Livak and Schmittgen, 2001). The basal expression level used in calculations was based on the expression levels of the control population.

### Respiration Rate Analysis

Thirty oysters that were 1-year-old were selected from the selected group and the control group to study the respiration rate. The oysters were cleaned with a brush prior to testing to eliminate other organisms (e.g., periphyton) that might affect estimates of respiration rate. Tests were conducted at normal water temperature (20°C) and under acute heat stress (35°C). Oysters were placed in plastic bottles full of water with oxygen, and air bubbles were removed. The seawater was mixed using a magnetic stirrer bar beneath the chamber. A needle-type fiber-optic microsensor oxygen sensor and temperature probe were connected to an oxygen transmitter that recorded the temperature and oxygen concentration of water every 3 s for 1.5 h. The slope of the decrease in oxygen concentration is the respiration rate. Respiratory rate results were normalized to remove differences among individuals.

### Field Experiment

Six-month-old oysters were used to examined thermal tolerance. These oysters from selected group and control group were subjected to heat shock treatment at 42°C for 1 h and removed to room temperature. The survival rate was recorded for 20 days. The survival rate of the selected group and the control group was tested in summer from June to October. In June 2020, 1-year-old oysters from the selected group and the control group were brought to the laboratory, and organisms visible on the surface were removed. Of a total of 300 oysters that were selected, 100 oysters were placed in each of three cages for both the selected group and the control group. The survival rate experiment was conducted in Jiaonan, Qingdao, Shandong.

### Statistical Analysis

All data were expressed as mean ± standard error of the mean. Statistical analyses were performed with SPSS 22.0 (IBM, NY, Untied States). Venny 2.1 was used to screen the candidate gene set, and the ggplot2 package in R was used to visualize the data. GraphPad Prism 8 was used to construct graphs. The threshold for statistical significance was *p* < 0.05.

## Results

### Transcriptomics Analysis

#### RNA-seq Data

In this study, transcriptomics analysis was performed in the selected and control groups of the second generation at ambient temperature for the larval stage. A total of 132.41 million reads were obtained from six samples, which had an average Q30 of 92.90% and average GC content of 40.15%, indicating that the data were high quality (Supplementary Figure 2). We obtained 4764 DEGs, among which 2568 were up-regulated genes and 2196 were down-regulated genes. The PCA plot showed transcriptional divergence between the selected group and the control group (Supplementary Figure 3); PC1 clearly separated the two groups and accounted for 38.22% of the variance.

**FIGURE 1:**
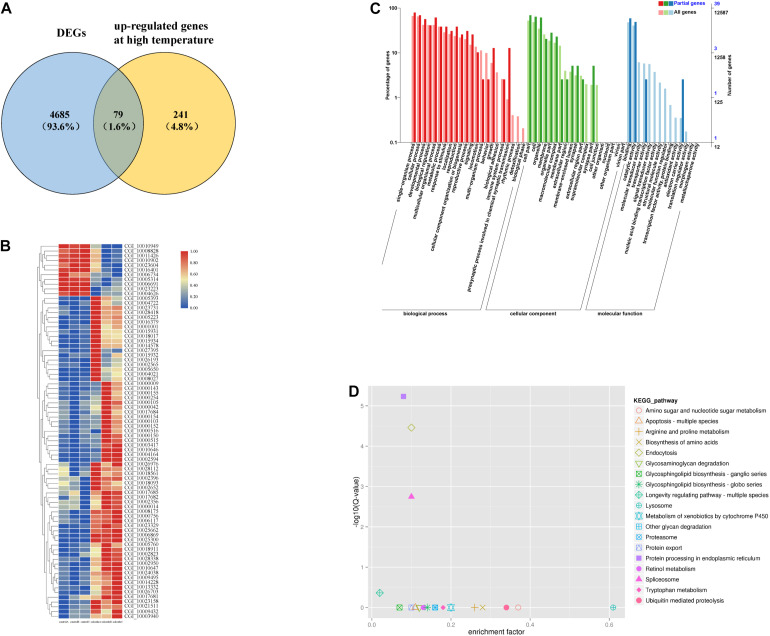
Among the high temperature response gens and DEGs, 79 genes were responsive to heat shock **(A)**. Hierarchical cluster analysis of 79 genes **(B)**. GO enrichment analysis of 79 genes **(C)**. KEGG enrichment analysis of 79 genes **(D)**.

Among the above DEGs between the selected group and the control group, 79 were heat-responsive genes (Zhang et al., 2012; Figure 1A). A hierarchical cluster analysis of the 79 genes showed that the selected group and the control group were significantly differentiated (Figure 1B).

#### GO and KEGG Pathway Analysis of the DEGs

Overall, 30 GO terms, including “unfolded protein binding,” “response to heat,” and “response to stress” were significantly enriched (Figure 1C, *p* < 0.05) among the 79 DEGs. There were 19 enriched KEGG pathways, four of which were significantly enriched (Figure 1D, *p* < 0.05). Five HSP family genes (*CGI_10002594*, *CGI_10002823*, *CGI_10003417*, *CGI_10010646*, and *CGI_10010647*) were enriched. Thirteen of the enriched GO terms contained these five HSP family genes; “ATPase activity” also contained four HSP family genes (*CGI_10002823* was the only gene not included). Four of the GO terms were related to stress response, four were related to energy metabolism, and six were related to growth and development (Table 1A). In addition, the 79 DEGs were enriched in three highly significant (*p* < 0.01) KEGG pathways related to protein processing, and each KEGG pathway contained the five HSP family genes (Table 1B). These five HSP family genes and the HSP-regulating gene *BAG4* (Zhu et al., 2015) were used as candidate genes to verify differences in gene expression between the selected group and the control group.

**TABLE 1A:** SNP statistics from HSPs which differentiated in gene structure and expression.

**SNP ID**	**ED**	**Mutation type**	**Location**
4949-1	0.7267	C/A	Intron
22585-2	0.7189	A/T	Upstream
22585-1	0.6809	C/T	Upstream
4949-2	0.6678	A/G	Intron
4823	0.697	A/C	Downstream
24709	0.6835	A/T	Intron
12734	0.6727	C/T	Downstream
13528	0.6613	A/T	Intron

**TABLE 1B:** Two SNPs differentiated in gene expression allele by genotyping.

**SNP ID**	**Location**	**Allele**	**Genotypes**
22585-1	Upstream	C>T	TT CT CC
24709	Intron	T>A	AA AT AT

#### Gene Expression in 1-Year-Old Oysters

**FIGURE 2:**
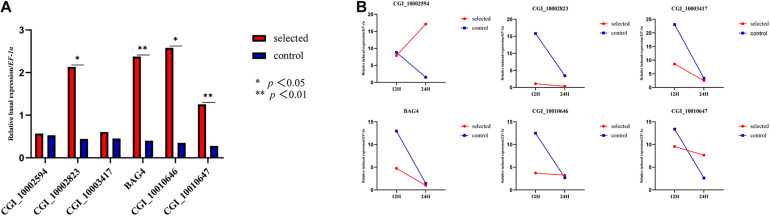
Relative basal expression of five HSP family genes and BAG4 **(A)**. Relative induced expression of five HSP family genes and BAG4 **(B)**.

One-year-old oysters from the selected group and the control group were subjected to heat shock for 24 h and sampled at 0, 12, and 24 h. The relative basal expression levels of *CGI_10002823*, *CGI_10010646, CGI_10010647*, and BAG4 were significantly different (*p* < 0.05) (Figure 2A). The relative induced expression of *CGI_10002594* in the selected group was up-regulated, and the expression of the control group was down-regulated between 12 and 24 h of heat shock; the remaining five genes had a small range of relative induced expression for the selected group, whereas the control group had a large expression range (Figure 2B).

### Whole-Genome Re-sequencing Analysis

#### Re-sequencing Data and SNP Screening

Whole-genome re-sequencing was performed on the selected group and the control group. A total of 6377816 SNPs were obtained for the selected group, and 6362948 SNPs were obtained for the control group; the average sequencing depth was 82× (Supplementary Figure 4).

**FIGURE 3:**
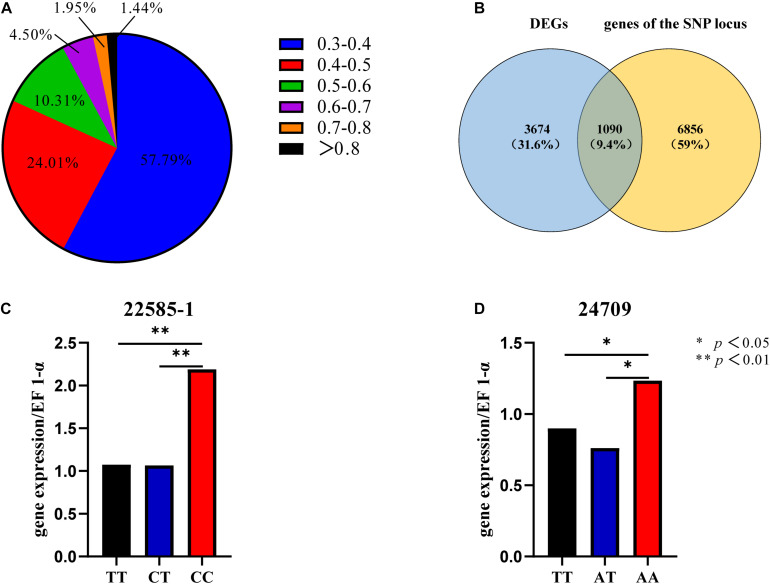
SNP frequency distribution **(A)**. Venn diagram of DEGs and genes of the SNP locus **(B)**. Gene expression levels of different genotypes of SNP 22585-1 **(C)**. Gene expression levels of different genotypes of SNP 24709 **(D)**.

The EDs for all SNPs were calculated, and SNPs with ED < 0.3 were filtered out. Frequency distribution diagrams were drawn (Figure 3A). The SNP frequencies for different ED classes were as follows: 0.3–0.4 (57.79%), 0.4–0.5 (24.01%), 0.5–0.6 (10.31%), 0.6–0.7 (4.50%), 0.7–0.8 (1.95%), and ED > 0.8 (1.44%). We focused exclusively on the SNPs in the genic region to identify the SNPs mediating gene expression. SNPs were in intronic (53%), upstream (20%), downstream (20%), synonymous (4%), and non-synonymous (3%) regions (Supplementary Figure 5A).

The top 5% of the SNPs with the largest ED was located in 7946 genes through Venny 2.1, among which 1090 were DEGs (Figure 3B). These SNPs were located in intronic (65%), upstream (14%), downstream (14%), synonymous (6%), and non-synonymous (1%) regions (Supplementary Figure 5B). The six HSP included three up-regulated genes (CGI_10004949, CGI_10022585, and CGI_10024709) and three down-regulated genes (CGI_10004823, CGI_10012734, and CGI_10013528). And the six HSPs had eight SNP which belonged to the top 5% of SNPs with the largest ED, The eight SNP were located in intronic, upstream, and downstream regions (Table 1A).

#### The Expression Level of Genes Which Differing in Structure and Expression

Sixty oysters that were 1-year-old were selected to verify the association between genotypes and gene expression. The genotypes of five candidate SNPs from three of the up-regulated candidate genes were detected by the SNaPshot technique. Two SNPs were divided into three genotypes (Table 1B).

The expression of the two genes that were successfully genotyped was further investigated by qPCR. For SNP 22585-1, the gene expression level of genotype CC was significantly higher than that of genotypes TT and CT, and the gene expression levels of CC, TT, and CT were significantly different (Figure 3C, *p* < 0.01). For SNP 24709, the gene expression level of genotype AA was significantly higher than that of genotypes TT and AT, and the gene expression levels of AA, TT, and AT were significantly different (Figure 3D, *p* < 0.05). The results showed that the dominant genotypes of SNP 22585-1 and SNP 24709 were CC and AA, respectively.

### Respiration Rate

**FIGURE 4:**
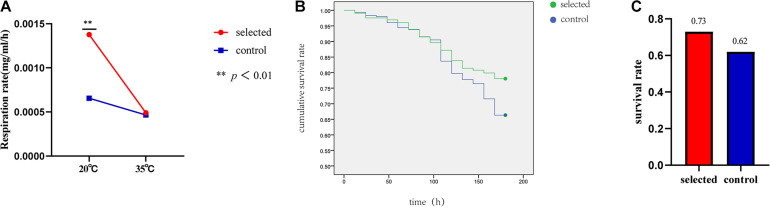
The respiration rate of selected group and control group at 20 and 35°C **(A)**. Summer survival in the selected and control groups **(B)**. The survival rates of selected group and control group after heat shock at 42°C for 1 h **(C)**.

Ten oysters were measured at one time during the experiment. The experiment was repeated three times and was completed within 6 h (10:00–16:00). The respiration rate of the two groups was significantly different (*p* < 0.01) when the seawater temperature was 20°C. The respiration rate of the selected group was 0.0012 mg/mL/h, and the respiration rate of the control group was 0.0006 mg/mL/h. At room temperature, the respiration rate of the selected group was much higher than that of the control group (Figure 4A). However, there was no significant difference in the respiration rates between the two groups when the seawater temperature was 35°C. The selected group respiration rate was 0.0005 mg/mL/h, and the control group respiration rate was 0.0004 mg/mL/h (Figure 4B).

### Survival Experiment

The survival rate after heat shock at 42°C for 1 h was used to evaluate the thermal resistance of oysters. The selected group exhibited a significantly higher survival rate (78.05%) during the recovery period compared with the control group (66.34%) following post-acute heat stress (*p* < 0.01) (Figure 4B). The summer survival rate in the field was estimated from June to October. The average survival rate of the selected group was 73% ranging from 69% to 78%; the average survival rate of the control group was 62% and ranged from 56 to 65% (Figure 4C).

## Discussion

The phenotypes, population structure, and gene expression level for thermotolerance were evaluated for the F_2_ generation. In 2017, we initiated a breeding program in which the surviving individuals were used as parents following acute thermal treatment of 42°C for 1 h. After two generations of selection, we used transcriptomic and re-sequencing to reveal the mechanism underlying the thermotolerance of the artificial selection population. We also evaluated the breeding effect by the respiration rate and the summer survival rate.

### Transcriptomics Analysis

The principal component analysis and hierarchical cluster analysis suggested that the selected group and the control group exhibited global divergence at the transcriptional level during the larval stage. The 79 heat-responsive genes of the 4674 DEGs may play significant roles in mediating the differentiation in thermotolerance between the selected group and the control group. Five HSP genes were significantly enriched in 14 GO terms and three KEGG pathways. The five genes that were enriched belonged to the HSP70 family. HSPs have been reported to play significant roles in the response to thermal stress in many organisms. Some studies have shown that unfolded protein binding is important for environmental stress resistance in shrimp (Song et al., 2019). Functional studies of crustacean HSPs, particularly HSP70s, have demonstrated their involvement in the activation of several immune pathways (Junprung et al., 2020). HSP70 and chaperonin TriC/CCT are involved in the folding of newly synthesized proteins (Frydman et al., 1994), whereas HSP70 and HSP90 are involved in the refolding of denatured proteins (Schneider et al., 1996). Response to methotrexate (MTX) was activates the AMPK pathway to increase oxidative metabolism and reduce cell proliferation (Papadopoli et al., 2020). Because the larvae were used for RNA-seq, pathways relating to growth development and energy metabolism were enriched, such as determination of adult lifespan, ATPase activity, microtubule-associated complex, mitochondrion, neurogenesis, ATP binding, and nucleus.

Previous studies have shown that these five HSP genes play major roles in the response to heat stress in oysters. For example, the expression of four heat-induced genes (*CGI_10002594*, *CGI_10002823*, *CGI_10010646*, and *CGI_10010647*) was 20 times higher than that of the control group, and the expression of *CGI_10003417* was five times higher than that of the control group (Zhang et al., 2012) under heat stress at 35°C. The expression of these four genes increased as the heat shock treatment progressed (Liu Y. et al., 2019). In addition to the transcriptional analysis of the larval stage, the expression of the five enriched HSPs and *BAG4* at ambient temperature and high temperature was further explored in the adult stage. Four genes (*CGI_1002823*, *BAG4*, *CGI_10010646*, and *CGI_10010647*) showed significantly higher expression in the selected group than in the control group at ambient temperature, which was consistent with the global divergence between the two groups at the larval stage. Five of the six genes exhibited low plasticity in the selected group in response to high temperature, suggesting that the selected groups had lower sensitivity to heat stress. Divergence in the plasticity between groups differing in environmental tolerance has also been reported in many other studies, suggesting that plasticity is an important component of stress tolerance (Liu S. et al., 2019).

### Whole-Genome Re-sequencing Analysis

SNPs from 1090 genes that were divergent in gene structure and gene expression were located in intronic, upstream, and downstream regions and accounted for 93% of all SNPs. SNPs located in introns were increased in these 1090 genes, whereas SNPs in upstream and downstream regions were reduced compared with other regions. This suggests that the SNPs that mediate differentiation in gene expression are more likely to be located in introns. The high number of SNPs in introns is inconsistent with previous studies in wild rice, which have shown that more SNPs were located in upstream regions (41%) compared with introns (17%) and downstream regions (20%) (Yang et al., 2020).

The two SNPs distributed in different regions of genes may indicate different regulatory mechanisms. The two SNPs of the two genes (*CGI_10022585* and *CGI_10024709*) were located in introns and upstream regions, respectively. SNP 22585-1 is located 2.1–2.2 kb upstream of the gene transcription start site, which is in the promoter region, and thus might affect the transcription process (Liu S. et al., 2019). Numerous polymorphisms associated with traits are localized in introns (Sharma et al., 2013; Liu et al., 2014; Tsai et al., 2014; Zhang et al., 2018). Three intronic SNPs were found to be associated with resistance to white spot syndrome virus in *Procambarus clarkii* (Zhang et al., 2021). Two intronic SNPs were also shown to affect growth traits in *O. mykiss* (Nazari et al., 2016). Five intronic SNPs were significantly correlated (*p* < 0.05) with glycogen content in Pacific oysters (Liu S. et al., 2019). SNP 24709 is located in the intronic region, and the AA genotype has a positive effect on thermotolerance. Individuals with favorable genotypic combinations comprised 8.3% of the two populations, indicating that molecular breeding could be conducted to improve thermotolerance using this favorable genotypic combination. The potential dominant genotypes of SNP 24709 and SNP 22585 were obtained, which were expressed in the intron and upstream region, respectively. These potential markers could be used for marker-assisted breeding.

### Oxygen Consumption Rate

Temperature is one of the most important abiotic factors affecting physiological responses in marine organisms (Windisch et al., 2011). In this study, the respiration rate of the selected group was significantly higher than that of the control group when the seawater temperature was 20°C. The two generations of selection led to physiological divergence between the two populations at room temperature.

Respiration rate is a commonly used physiological index for monitoring the dynamic response to stress. In previous studies, the respiration rate significantly differed between the selected and control populations when the temperature increased from 35 to 38°C (*p* < 0.05) (Ding et al., 2020). The respiration rate of *C. gigas angulata* ware significantly higher than that of *C. gigas* at 32, 36, and 40°C (Ghaffari et al., 2019). The respiration rate was significantly different between *Haliotis discus hannai* (DD) and hybrid offspring (*H. discus hannai* ♀ × *Haliotis fulgens*♂, DF) at 28°C; specifically, DD appears more vulnerable to thermal stress than DF (Shen et al., 2020). In this study, the effects of the selected group after two generations of selection were assessed. In normal temperature (20°C) seawater, the respiration rate of the selected group was significantly higher than that of the control group, which reflected the effect of artificial selection. At 35°C, the respiration rate of the two groups decreased with different magnitude, which reflected divergence in the thermotolerance of the two groups.

### Survival Rate

The survival rate was used to assess the thermotolerance of oysters following acute heat stress. The survival rate of the F_2_ thermotolerance oysters after exposure to acute heat stress at 42°C in the selected population was significantly higher than that of the control population, suggesting that the thermotolerance of selected oysters had improved. The survival rate was even more improved compared with F_1_ thermotolerance oysters (Ding et al., 2020). Heritability for survival during summer was high, which suggested that selective breeding could effectively improve the thermotolerance of Pacific oyster spat (Dégremont et al., 2007). The summer survival rate in the field was used to verify the thermotolerance of the selected group. The summer survival rate of the selected group was 73%, and that of the control group was 62%. The results are consistent with our previous study showing that the summer survival rate of the F_1_ thermotolerance oysters (74.19%) was significantly higher compared with the control (62.33%) (Ding et al., 2020). This result is also consistent with the results of a previous study showing significantly higher survival of selected populations during summer at three different sites in the F_2_ and F_3_ generations in 6-month-old Pacific oysters (Dégremont et al., 2010). The selected population showed a higher survival rate compared with the control population, indicating a positive response to selection for survival. Mortality in the wild may be caused by several environmental factors. Considering that mass mortality did not occur in this region during the testing period, the slight difference in the survival rate may not fully reflect the thermotolerance of the two groups.

## Data Availability Statement

The data presented in the study are deposited in the Sequence Read Archive (SRA) repository, accession numbers PRJNA697824 and PRJNA694496.

## Author Contributions

LL and RC conceived the experiment. RC and LW collected the oysters and performed the spawning. YT performed the experiment and collected and analyzed the data. YT, LL, RC, HQ, and YP wrote and edited the manuscript. All authors contributed to the article and approved the submitted version.

## Conflict of Interest

The authors declare that the research was conducted in the absence of any commercial or financial relationships that could be construed as a potential conflict of interest.
