# Supplementary material for: Transcriptomics Analysis and Re-sequencing Reveal the Mechanism Underlying the Thermotolerance of an Artificial Selection Population of the Pacific Oyster
Source: Front Physiol. 2021 Apr 22;12:663023. doi: 10.3389/fphys.2021.663023 (PMC8100323; doi:10.3389/fphys.2021.663023)
Supplement: Supplementary file 1 [file Table_1.pdf]

Table. S1A

Category	GO term	*P* -value	Gene ID
response to methotrexate	GO:0031427	1.99E-10	CGI_10010646<br>CGI_10003417<br>CGI_10010647<br>CGI_10002594<br>CGI_10002823
unfolded protein binding	GO:0051082	5.65E-09	CGI_10003417<br>CGI_10002594<br>CGI_10002823<br>CGI_10027395<br>CGI_10010646<br>CGI_10010647
response to heat	GO:0009408	1.11E-07	CGI_10010646<br>CGI_10002823<br>CGI_10002594<br>CGI_10010647<br>CGI_10027395<br>CGI_10003417
response to starvation	GO:0042594	2.25E-07	CGI_10010647<br>CGI_10010646<br>CGI_10002823<br>CGI_10003417<br>CGI_10002594
protein folding	GO:0006457	2.95E-07	CGI_10010647<br>CGI_10027395<br>CGI_10003417<br>CGI_10010646<br>CGI_10002823

			CGI_10002594
			CGI_10002823
			CGI_10005650
			CGI_10010647
lipid droplet	GO:0005811	1.54E-06	CGI_10010646
			CGI_10002594
			CGI_10027395
			CGI_10003417
			CGI_10002594
			CGI_10003417
			CGI_10015932
			CGI_10002823
			CGI_10008027
determination of adult lifespan	GO:0008340	3.36E-06	CGI_10004164
			CGI_10015934
			CGI_10010647
			CGI_10003940
			CGI_10010646
			CGI_10027395
			CGI_10010647
ATPase activity	GO:0016887	1.55E-05	CGI_10003417
			CGI_10010646
			CGI_10002594
			CGI_10002823
			CGI_10004164
			CGI_10002594
response to stress	GO:0006950	2.28E-05	CGI_10003417
			CGI_10010647
			CGI_10010646

			CGI_10027395
microtubule associated complex	GO:0005875	4.60E-05	CGI_10003417<br>CGI_10002594<br>CGI_10002823<br>CGI_10027395<br>CGI_10010646<br>CGI_10010647<br>CGI_10025300
mitochondrion	GO:0005739	4.83E-05	CGI_10003417<br>CGI_10027395<br>CGI_10002594<br>CGI_10010646<br>CGI_10010647<br>CGI_10002823
neurogenesis	GO:0022008	0.00022023	CGI_10002823<br>CGI_10003417<br>CGI_10002594<br>CGI_10010647<br>CGI_10018093<br>CGI_10010646
ATP binding	GO:0005524	0.001648108	CGI_10010646<br>CGI_10002823<br>CGI_10002594<br>CGI_10003417<br>CGI_10010647<br>CGI_10011426<br>CGI_10027395
nucleus	GO:0005634	0.026615088	CGI_10002594<br>CGI_10003417

			CGI_10002823<br>CGI_10010646<br>CGI_10027395<br>CGI_10023329<br>CGI_10010647<br>CGI_10028112

Table. S1B

Category	KEGG pathway	*P* -value	Gene ID
Protein processing in endoplasmic reticulum	ko04141	3.07E-07	CGI_10001001<br>CGI_10002594<br>CGI_10002823<br>CGI_10003417<br>CGI_10010646<br>CGI_10010647<br>CGI_10027395
Endocytosis	ko04144	1.82E-06	CGI_10002594<br>CGI_10002823<br>CGI_10003417<br>CGI_10003940<br>CGI_10005650<br>CGI_10010646<br>CGI_10010647

Spliceosome	ko03040	9.46E-05	CGI_10002594<br>CGI_10002823<br>CGI_10003417<br>CGI_10010646<br>CGI_10010647
