# Supplementary material for: Transcriptomics Analysis and Re-sequencing Reveal the Mechanism Underlying the Thermotolerance of an Artificial Selection Population of the Pacific Oyster
Source: Front Physiol. 2021 Apr 22;12:663023. doi: 10.3389/fphys.2021.663023 (PMC8100323; doi:10.3389/fphys.2021.663023)
Supplement: Supplementary file 2 [file Image_1.pdf]

Figure S1

Gene id	Primer sequence F	Primer sequence R
CGI_10002594	TCAGCCAAGGACAAGAGCACAG	TCGGCCTCGTTCACCATTCTCT
CGI_10002823	GCTGTGGCTTATGGAGCTGCTG	TCCTGCCGTTTCAATGCCCAAA
CGI_10003417	AATGCCCAAGAAATCGCC	GACCAATTAGTCGTTTAGCGTCAA
CGI_10026703	TCAACAACAGCAGTATCAGGCGAAA	CCGACTCTACACTCTCACTCCTCTG
CGI_10010646	CACCGTTCCTGCCTACTT	GCTGTGGGTTCATTTACTATC
CGI_10010647	CACCGTTCCTGCCTACTT	GCTGTGGGTTCATTTACTATC
CGI_10022585	ATCAGAAAAGCGGTTACG	TTCCTCTTGGTCATCAGTG
CGI_10024709	GTTGTTCGTATGTGCTATTGAG	CATAACCAGAATACATCAGCC
