# Supplementary material for: Transcriptomics Analysis and Re-sequencing Reveal the Mechanism Underlying the Thermotolerance of an Artificial Selection Population of the Pacific Oyster
Source: Front Physiol. 2021 Apr 22;12:663023. doi: 10.3389/fphys.2021.663023 (PMC8100323; doi:10.3389/fphys.2021.663023)
Supplement: Supplementary file 3 [file Image_2.pdf]

Figure S2

ID	Clean Reads	≥Q30	GC Content
Selected A	23941716	92.90%	39.96%
Selected B	20502956	92.98%	39.52%
Selected C	22183461	93.09%	38.43%
Control A	21776617	93.46%	40.87%
Control B	21642163	93.88%	41.06%
Control C	22367076	93.03%	41.07%
