# Supplementary figures and images for: Transcriptomics Analysis and Re-sequencing Reveal the Mechanism Underlying the Thermotolerance of an Artificial Selection Population of the Pacific Oyster
Source: Front Physiol. 2021 Apr 22;12:663023. doi: 10.3389/fphys.2021.663023 (PMC8100323; doi:10.3389/fphys.2021.663023)

Figure S3

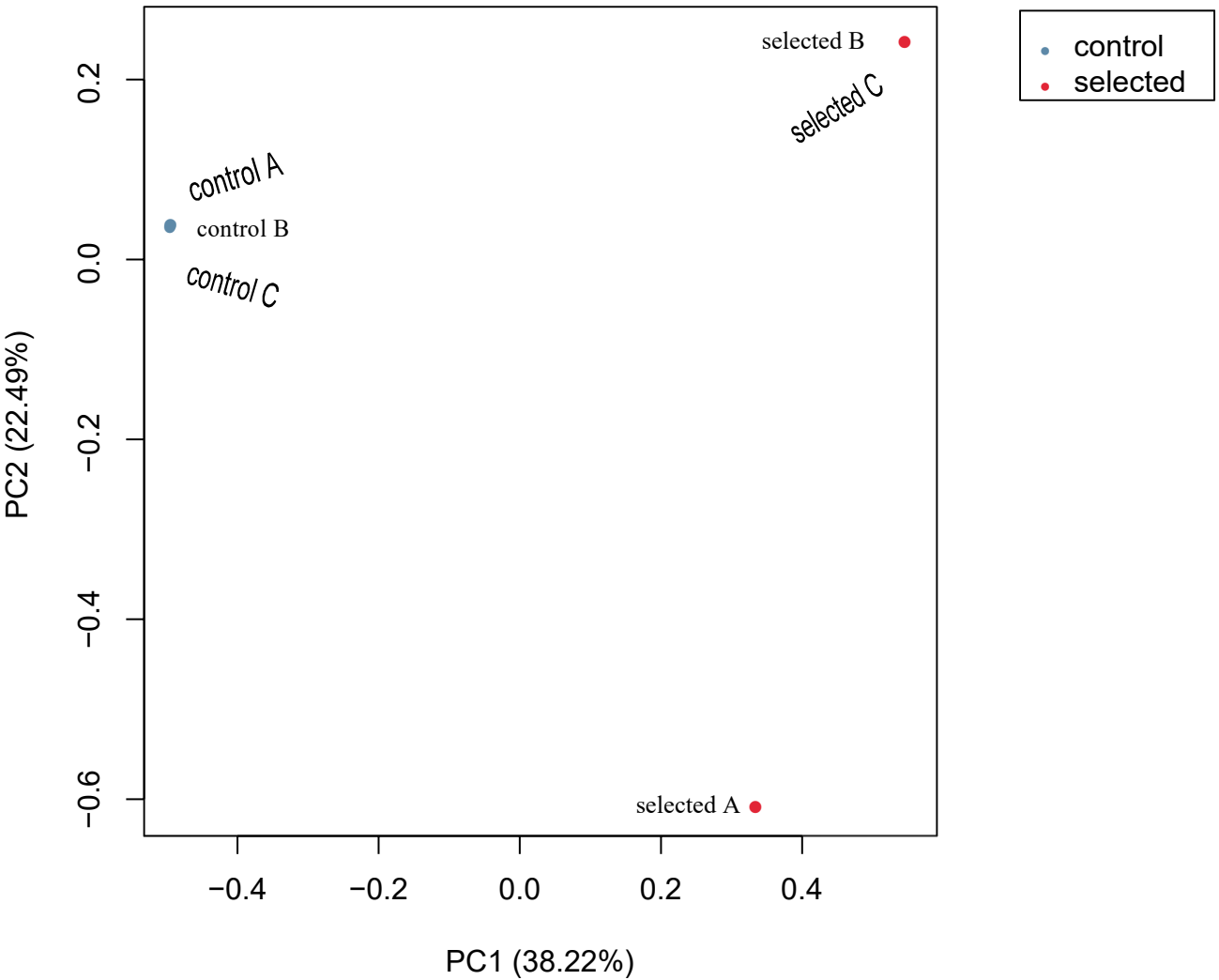

Supplement: Supplementary file 4 [file Image_3.pdf]

Figure S5A

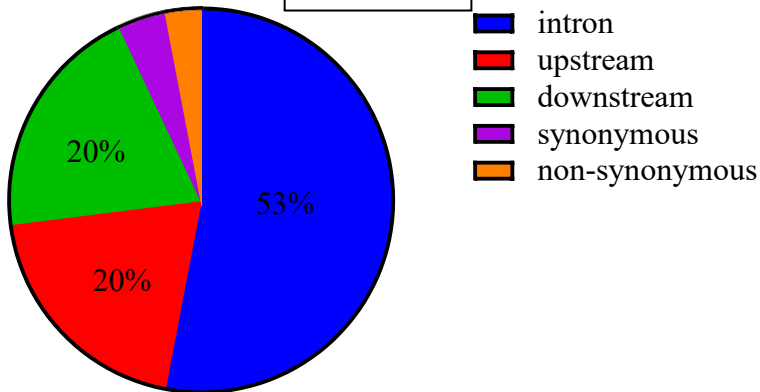

Figure S5B

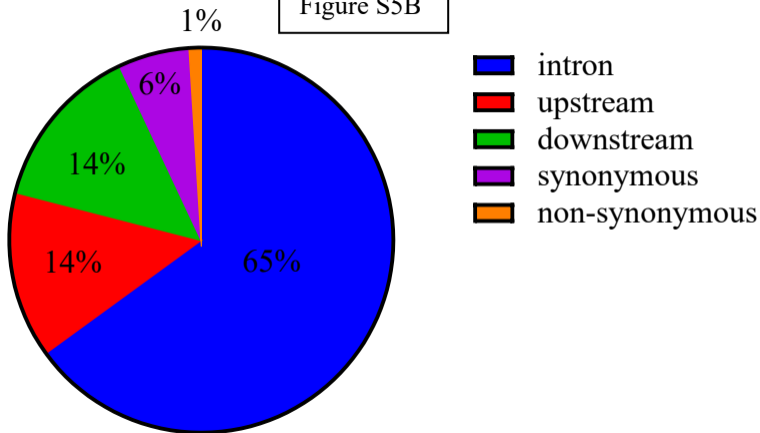

Supplement: Supplementary file 6 [file Image_5.pdf]
