# Supplementary material for: Transcriptomics Analysis and Re-sequencing Reveal the Mechanism Underlying the Thermotolerance of an Artificial Selection Population of the Pacific Oyster
Source: Front Physiol. 2021 Apr 22;12:663023. doi: 10.3389/fphys.2021.663023 (PMC8100323; doi:10.3389/fphys.2021.663023)
Supplement: Supplementary file 5 [file Image_4.pdf]

Figure S4

ID	SNP number	Mapped(%)	Ave_depth	Genome coverage
selected	6377816	90.07％	84X	99.01％
control	6362948	90.03％	80X	98.94％
